# Delayed Asthmatic Response to Allergen Challenge and Cytokines Released by Nonspecifically Stimulated Blood Cells

**DOI:** 10.1155/2013/496208

**Published:** 2013-02-13

**Authors:** Zdenek Pelikan

**Affiliations:** Allergy Research Foundation, Effenseweg 42, 4838 BB Breda, The Netherlands

## Abstract

*Background*. Bronchial asthma patients can develop various asthmatic response types following bronchial allergen challenge, such as immediate (IAR), late (LAR), dual late (DLAR), or delayed (DYAR), due to different immunologic mechanisms. The DYAR, recorded in 24 patients, beginning between 26 and 32 hrs and lasting up to 56 hrs after the bronchial allergen challenge, differs from the IAR, LAR, and DLAR in clinical, diagnostic, and immunologic aspects. *Objective*. To investigate amounts of particular cytokines released by the blood cells after an additional nonspecific stimulation with Phorbol 12-myristate 13-acetate (PMA) during the DYAR. *Methods*. In 24 patients, the repeated DYAR was supplemented with determination of cytokines both in the nonstimulated plasma and in the supernatants of the blood cells stimulated with PMA before and up to 72 hours after the bronchial challenge, by means of enzyme-linked immunoassay. *Results*. No significant changes of the prechallenge cytokine concentrations in the non-stimulated serum were recorded in the DYAR patients as compared with the healthy subjects. The DYAR was accompanied by significantly increased postchallenge concentrations (*P* < 0.05) of IL-2, IL-8, IL-12p70, IL-13, IL-18, IFN-**γ**, G-CSF, TNF-**α**, and TGF-**β**, while decreased concentration of IL-7 (*P* < 0.05) in the nonstimulated plasma. The significantly increased postchallenge concentrations of IL-2, IL-8, IL-12p70, IL-13, IL-18, IFN-**γ**, TNF-**α**, and TGF-**β** were released by peripheral blood cells after stimulation with PMA, as compared with both their prechallenge concentrations and with the PBS control values. *Conclusions*. These results would support evidence for an important role of the Th1 cells, neutrophils, monocytes, and probably also NK cells in the immunologic mechanism(s) leading to the development of the clinical DYAR. Nevertheless, an additional role of macrophages, endothelial and epithelial cells in these mechanisms cannot be even excluded.

## 1. Introduction 

Allergic bronchial asthma is a multifaceted disorder, where various immunologic mechanisms can participate [1–8]. The causal role of immediate hypersensitivity including IgE antibodies, mast cells, eosinophils, and Th_2 _lymphocytes, in this disorder has been established [1–11]. Knowledge of the role of other hypersensitivity mechanisms in this condition remains still deficient [1–4, 7–18]. 

Bronchial asthma patients can respond to allergen challenge by various types of asthmatic response, such as immediate (IAR), late (LAR), or dual late (DLAR) responses [5, 7, 8, 10, 11, 18–23], having been investigated extensively from various clinical, immunologic and pharmacologic points of view [5, 7–39]. 

In some patients examined at our clinic, an unusual asthmatic response appearing 26–56 hours after the bronchial challenge with inhalant allergens was recorded [40, 41]. This response, displaying clinical and immunologic features different from those of the IAR and of the LAR, was designated as “delayed asthmatic response” (DYAR) [40, 41]. 

Cytokines representing very important components of the immunologic mechanism(s) execute a number of intracellular, extracellular, and intercellular activities, such as stimulating, inhibiting, and chemotactic effects as well as signal transmission among various cell types [1, 2, 6, 17, 18, 21–33]. From this point of view, the cytokines are closely related to various cell types participating on various levels and during various stages of the immunologic events. This relation has two basic aspects: a morphologic aspect concerning the production of particular cytokines by certain cell types and a functional aspect concerning the specific effects of particular cytokines on various target cell types [1, 2, 6]. The cellular sources of particular cytokines, being a very complex and continuously updated topic, are actually outside the scope of this study and are presented in detail in a number of excellent reviews [6]. 

The purpose of this study was to investigate: (1) the intracellular cytokine potential which can be released after the “*in vitro”* nonspecific stimulation of the blood cell in patients developing the DYAR in comparison with the patients demonstrating IAR, LAR, or DLAR as well as with healthy subjects; (2) the ratio between the concentrations of cytokines released into the peripheral blood “*in vivo*” during the DYAR and the amounts of intracellular cytokines released by “*in vitro*” nonspecific cell stimulation; (3) the activation degree of the particular blood cell types during the DYAR and that caused by the *“in vitro*” nonspecific stimulation.

## 2. Material and Methods

### 2.1. Patients

**Figure 1:**
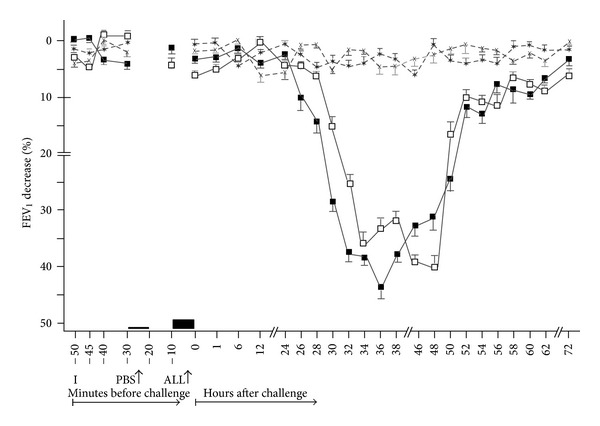
Delayed asthmatic response to allergen challenge (DYAR) and phosphate buffered saline (PBS) control challenge. The mean percentage changes in the FEV_1_ values calculated from 24 DYARs and 24 PBS control challenges; (■): the initial DYAR; (□): the repeated DYAR; (∗): the initial PBS; (×): the repeated PBS; I: initial (baseline) values; ALL: allergen challenge; PBS: phosphate buffered saline; Bars: means ± SEM.

Twenty-four patients with bronchial asthma examined at our Department of Allergology and Immunology, Inst. Med. Sci “De Klokkenberg”, Breda, The Netherlands), and developing DYAR (Figure 1) after the bronchial allergen challenge (BPT), volunteered to participate in this study. 

These patients, 21–48 years of age, demonstrating reversible bronchial constriction alternating with symptom-free periods, showed pulmonary function without any restrictive changes (Table 1). They had no airway infections and did not use oral corticosteroids or immunotherapy. They were examined by routine diagnostic procedure, serving also as an inclusion-exclusion criteria, consisting of various diagnostic parameters (Table 2) and including also 43 BPTs with inhalant allergens (Table 3) and 24 PBS control challenges. All BPTs were performed in a period without manifest bronchial complaints, outside the allergen-relevant season and during hospitalization.

Inhalation corticosteroids (*n* = 6) and long-acting *β*_2_-sympathomimetics (*n* = 8) were withdrawn 4 weeks, cromolyn (*n* = 3), nedocromil sodium (*n* = 5) and leukotriene-antagonists (*n* = 2) 2 weeks, and other treatments 48 hours prior the BPTs. If the postchallenge FEV_1_ values decreased with 50% or more with respect to the predicted values, the patients were treated with inhalation of 200–400 mcg Salbutamol.

In the DYAR patients and all control subjects, a single determination of various cytokines both in the nonstimulated serum (Table 4) and those released from peripheral blood cells after an “*in vitro*” stimulation with Phytohemagglutinin (PHA), Phorbol 12-myristate 13-acetate (PMA), and N-formylmethionyl-leucyl-phenylalanine (fMLP) (Table 5) was performed.

In the DYAR patients, the BPTs and PBS controls were repeated 2–6 weeks later. The repeated DYARs and PBS controls (Figure 1) were supplemented with recording of the cytokines in the nonstimulated serum and cytokines released by peripheral blood cells after the “*in vitro*” stimulation with PMA at 1, 12, 24, 36, 48, 56, and 72 hours after the challenge (Table 6). The local ethical committee (IRB-MCK) approved this study and an informed consent in written was obtained from all participants. 

### 2.2. Control Subjects

The 21 asthmatics demonstrating an isolated IAR, 17 asthmatics developing an isolated LAR, 18 asthmatics showing DLAR, and 26 healthy subjects volunteered to participate as control subjects in this study (Tables 1–5).

### 2.3. Allergens

Dialyzed and lyophilized allergen extracts (Allergopharma, Reinbek, Germany) diluted in PBS were used in concentrations of 100–500 BU/mL for skin tests and 1000–3000 BU/mL for BPTs (Table 3). The concentrations recommended by the manufacturer were 500 BU/mL for skin tests and 5000 BU/mL for the BPTs [40].

### 2.4. Skin Tests

Scratch tests with allergenic extracts in concentrations of 500 BU/mL were evaluated after 20 minutes. If they were negative, intracutaneous tests in concentration of 100 BU/mL and then 500 BU/mL were performed and evaluated 20 minutes, 6, 12, 24, 36, 48, 72, and 96 hours after the intradermal injection. A skin wheal reaction (>7.0 mm in diameter) occurring after 20 minutes was qualified as positive immediate skin response, skin infiltration appearing between 6 and 12 hours as a late skin response and skin induration observed later than 48 hours was considered a delayed skin response [40]. 

### 2.5. Bronchial Provocation Tests (BPT)

The BPTs were performed by means of spirometry (Spirograph D-75, Lode, Groningen, The Netherlands) recording the FVC and FEV_1_ values. The allergen extracts and PBS were inhaled in the form of an aerosol using the Wiesbadener Doppel-Inhalator at an airflow of 10 L/min. The nebulizer output was 0.12–0.14 mL/min and the aerosol particles were of a median mass diameter of 2.8–3.6 *μ*.

The BPTs, being a modification of the European standard [42], were performed according to the following schedule: (1) initial (baseline) values recorded at 0, 5, and 10 minutes; (2) PBS control values recorded at 0, 5, and 10 minutes after a 10-minute PBS inhalation; (3) inhalation of allergen aerosol for 2 × 5 minutes, with inserted spirometric value measurement, followed by the recording of the FEV_1_ and FVC values at 0, 5, 10, 20, 30, 45, 60, 90, and 120 minutes and the every hour up to 12th hour and every second hour during the 22nd and 38th and the 46th and 56th hour intervals. The control challenge with PBS was performed using the same schedule as that applied to the BPTs with allergens. A 5-day interval has always been inserted between the consecutive tests [40].

### 2.6. Supplementary Parameters

Th_1_/Th_2_ ratio (%)  values in peripheral blood were determined by flow-cytometry and the intracellular IFN-*γ* and IL-4 in the peripheral blood mononuclear cells (PBMCs) stimulated with PMA were estimated by immunoassay ELISA-kits, as described previously [41].

### 2.7. Determination of Cytokines

Two series of venous blood were collected in all cases. Samples of 3 mL blood were, after clotting, centrifuged at 3000 ×g for 10 minutes and serum aliquots were stored at −70°C. Samples of 6 mL heparinized blood were centrifuged at 1800 g for 5 minutes at 4°C and plasma aliquots were stored at −70°C. The unseparated blood cells were washed in RPMI 1640 (Sigma-Aldrich, St. Louis, USA), centrifuged at 2000 ×g for 10 minutes at 4°C and resuspended in RPMI 1640 supplemented with penicillin (100 IU/mL), streptomycin (100 *μ*g/mL), and L-glutamine (2 mmol/L) at a concentration of 5 × 10^6^ leukocytes/mL. The cell viability was confirmed by trypan blue dye exclusion. The cell suspension was divided into 4 equal portions. Three portions, stimulated with 10 *μ*g/mL of PHA (Wellcome Diagnostics, Dartford, UK), PMA (Sigma-Aldrich) in a concentration of 50 ng/mL and 1 *μ*g/mL ionomycin or 1 *μ*mol/L of fMLP (Sigma-Aldrich), and the nonstimulated control portion, were cultured for 24 hours at 37°C under 5% CO_2_ in a humidified incubator. The supernatants were collected by centrifugation at 2000 ×g for 15 minutes and aliquots were stored at −70°C. The cytokines in the supernatants as well as in the nonstimulated plasma were estimated by commercial immunoassay (ELISA, EIA) kits, following the manufacturers' recommendations. All measurements were performed in duplicate. The detection limits in pg/mL are reported in brackets. The cytokines Il-1*β*  (1.0), IL-2 (<3.0), IL-3 (31.2), IL-5 (3.0), IL-6 (4.0), IL-7 (0.1), IL-10 (<3.0), IL-13 (<3.0), GM-CSF (<3.0), G-CSF (0.8), TNF-*α*  (6.0), and TGF-*β* (6.0) were measured using the R & D System (Minneapolis/MN, USA) kits, IL-4 (0.6), IL-8 (1.3), IL-12p70 (2.1), IL-18 (9.2), and IFN-*γ*  (1.0) by Bender MedSystems (Wien, Austria), and IL-12p40 (3.9) by Becton Dickinson (San Jose, USA) kits. The interassay as well as intraassay coefficients of variations for these kits were less than 10%.

### 2.8. Statistical Analysis

The initial and repeated DYAR and PBS controls were statistically evaluated by means of fitting polynomials to the mean curves over time; eight time points within 120 minutes and twenty-four time points up to 56 hours after the challenge. The hypotheses were tested by the generalized multivariate analysis of the variance model (MANOVA) [43]. 

The postchallenge cytokine values recorded at each of the time points during the repeated DYAR and PBS controls in individual patients were compared with their prechallenge values and statistically analyzed by Wilcoxon matched-pair signed rank test. The mean postchallenge values of individual cytokines measured at each of the time points during the repeated DYARs were compared with the corresponding mean PBS values and statistically evaluated by Mann-Whitney *U* test. A *P* value < 0.05 was considered to be statistically significant.

## 3. Results

### 3.1. Initial DYAR

The DYAR beginning between 26 and 32 hours, reaching its maximum between 32 and 48 hours and resolving within 56 hours after the allergen challenge (Figure 1) was significantly positive both in comparison of the postchallenge with the prechallenge FEV_1_ values (*P* < 0.01) and with the PBS values (*P* < 0.001). No significant differences in the appearance of DYAR were found with respect to the individual allergens (*P* = 0.21) (Table 3).

The DYAR was associated with increased blood leukocyte, neutrophil, and lymphocyte counts (Table 1), changed Th_1_/Th_2_ cell ratio in peripheral blood in favour of Th_1_ cells (*P* < 0.01), increased intracellular concentration of IFN-*γ* (*P* < 0.05) but not of IL-4 (*P* > 0.05) (Table 1), decreased bronchial histamine threshold in 63% (Table 1), positive immediate skin response in 42%, and positive delayed skin response in 50% (Table 2).

### 3.2. Repeated DYAR

The repeated DYAR was also significantly positive both comparing the postchallenge values with the prechallenge FEV_1_ values (*P* = 0.001) and with the corresponding PBS values (*P* = 0.001) (Figure 1). No statistically significant differences were found between the initial and the repeated DYARs (*P* = 0.17).

### 3.3. Prechallenge Cytokine Concentrations in Nonspecifically Stimulated Blood Cells

The prechallenge concentrations of some cytokines in the nonstimulated serum of the DYAR patients, such as IL-2, IL-6, IFN-*γ*, and GM-CSF, differed slightly, but not significantly, from those recorded in the healthy subjects as well as from the control asthmatics demonstrated IAR, LAR, and DLAR (Table 4).

The prechallenge cytokine concentrations released by the “*in vitro*” stimulated peripheral blood cells into supernatants of DYAR patients differed from those measured both in the healthy subjects and in the control asthmatics developing other asthmatic response types (Table 5). The mean cytokine concentrations released after stimulation with PHA, PMA, or fMLP did not differ significantly with respect to the particular agent, although the fMLP values were slightly lower than the PHA or PMA values (Table 5). No significant differences (*P* > 0.05) were found between the concentrations of individual cytokines recorded in the nonstimulated serum (Table 4) and those in the nonstimulated plasma (Table 6) of the DYAR patients.

### 3.4. Postchallenge Cytokine Concentrations in Nonspecifically Stimulated Blood Cells

**Figure 2:**
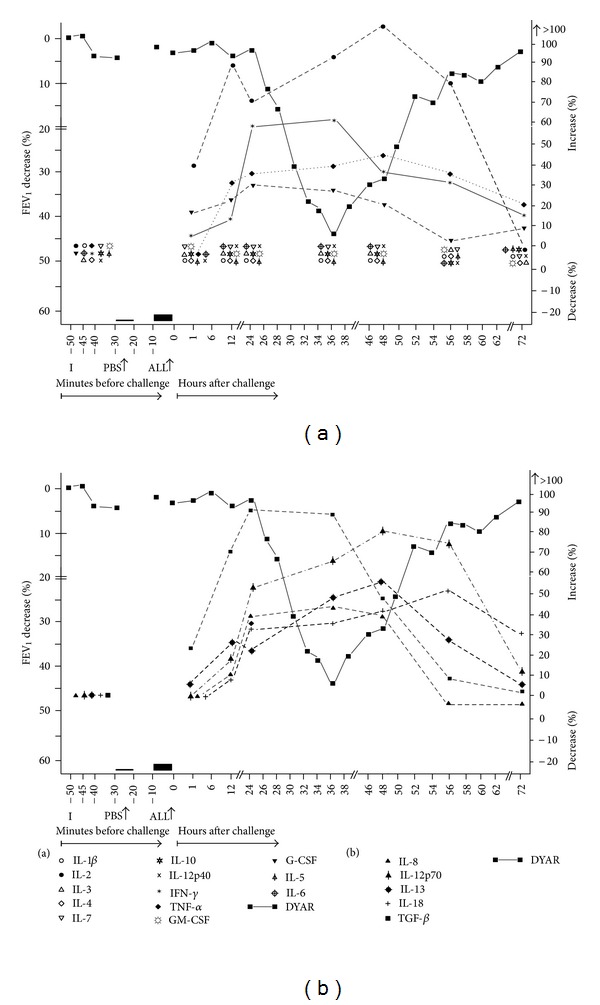
The repeated delayed asthmatic response to allergen challenge (DYAR) and the mean percentage changes in the concentrations of particular cytokines released from isolated blood cells stimulated with PMA; I: initial (baseline) value; ALL: allergen challenge; PBS: phosphate buffered saline.

The DYAR was associated with significant changes (*P* = 0.032–0.045) of the postchallenge cytokine concentrations, as compared both with their prechallenge values and with the corresponding PBS control values, as follows: (i) In the nonstimulated plasma: increased concentrations of IL-2, IL-8, IL-12p70, IL-13, IL-18, IFN-*γ*, G-CSF, TNF-*α*, and TGF-*β*; a decreased concentration of IL-7; (ii) In the supernatants of the blood cells stimulated with PMA: increased concentrations of IL-2, IL-8, IL-12p70, IL-13, IL-18, IFN-*γ*, TNF-*α*, and TGF-*β* (Table 6, Figures 2(a) and 2(b)). Most of these changes occurred 24–56 hours after the allergen challenge, which is the time of the maximal DYAR performance (Figure 1). No significant changes in the plasma (*P* > 0.05) or supernatants (*P* > 0.1) were found during the PBS controls. 

### 3.5. Control Subjects

In the control patients demonstrating IAR, LAR or DLAR, as compared with healthy control subjects, significant changes in the concentrations of various cytokines both in the nonstimulated serum (Table 4) and in the supernatants of the “*in vitro*” stimulated blood cells (Table 5) were recorded. These changes differed significantly from those measured in the DYAR patients. 

## 4. Discussion 

The DYAR differs from the IAR, LAR, and DLAR in clinical course, immunologic features and pharmacologic modulation [10, 11, 19, 20, 40, 41]. The DYAR is associated with profiles of eicosanoids, adhesion molecules, and cytokine in peripheral blood different from those accompanying the IAR, LAR, and DLAR, suggesting involvement of different immunologic mechanisms in the particular asthmatic response types [1, 2, 5–18, 21–41, 44, 45]. 

Cytokines, with their manifold biologic effects, represent very important components of the immunologic mechanisms [1, 2, 6, 9, 13, 23–39]. They participate in various stages of the immunologic mechanisms not only as intercellular signal transmitting factors, but also as a regulatory, for example, stimulating, inhibiting, and chemotactic, factors for most cell types involved in immunologic processes [1, 2, 6–9, 11, 13–18, 21–39]. Additionally, various cytokines can act by a synergistic or antagonistic manner among them. Their role in the allergic bronchial asthma and their significance for the diagnostic conclusions have already been investigated from various points of view [1, 2, 5–9, 13–18, 21–39]. 

In most of these studies, a single measurement of various cytokines in the asthma patients was performed [13, 14, 17, 26, 32, 35, 39]. In some of the studies, the cytokines were determined in the bronchoalveolar lavage (BAL) fluid or (induced) sputum after the segmental allergen challenge [8, 9, 15, 18, 21, 22, 24, 25, 27–30, 34]. In only few papers, the cytokines were studied in peripheral blood (PB) during the particular types of asthmatic response, such as IAR, LAR, or DLAR, due to the bronchial challenge with inhaled allergens (BPT) [18, 21, 23]. Moreover, in these infrequent studies, the intracellular cytokines were usually determined in the supernatants of the isolated blood cell after their “*in vitro*” stimulation with nonspecific agents, such as PHA, PMA, fMLP, or LPS [9, 13, 14, 17, 21, 25–33, 35].

The papers dealing with changes of the cytokine concentrations in peripheral blood accompanying the particular asthmatic response types caused solely by the allergens inhaled during the BPTs are relatively unique [21, 45, 46]. 

Results concerning the changes of particular cytokines in patients with allergic bronchial asthma relatively vary. These variations could be caused by several factors, such as variations in the asthmatic patient populations; different bronchial asthma phenotypes; intermittent or concomitant conditions; unexpected or uncontrolled additional allergen exposure; extent of the diagnostic procedures; differences in the allergen challenge techniques and techniques of material collection and processing; stage of the bronchial asthma and the functional stage of particular cell types in various media at the time of material collection; physiologic variations in the cytokine concentrations in the same medium as well as among the media themselves. 

One of the very important aspects and cause of some differences among the results reported by various papers concerns the mode of stimulation of the airway and circulating cells to release their cytokines. The cells stimulated by the “*in vivo”* inhaled allergen released distinctly lower cytokine amounts than those released after “*in vitro*” stimulation.The “*in vitro*” stimulation by a nonspecific but very powerful stimulating agent results usually in a release of the almost complete intracellular cytokine potential, including even those portions which would not be released upon natural allergen inhalation [45]. It can, therefore, be presumed that the cytokine amounts released after an “*in vitro*” stimulation with nonspecifically acting compounds, such as PHA, PMA, fMLP, or LPS, will be distinctly higher than those released by the natural allergen exposure. This fact may be confirmed by our current results of higher cytokine concentrations released into supernatants after the “*in vitro*” stimulation with PMA than cytokine amounts recorded at corresponding time intervals in either nonstimulated plasma (Table 6) or nonstimulated serum (Table 4) as well as by our previous results [45]. Moreover, no significant differences were found in the cytokine concentrations between the serum and the plasma (Tables 4 and 6). Unfortunately, the possible discrepancy between the cytokine amounts and ratio released by the “*in vivo*” inhaled allergen and those released after the “*in vitro*” stimulation of the isolated BAL or peripheral blood cells by nonspecific stimulating agents has not yet been sufficiently investigated. 

An additional aspects concerns the releasing capability of the particular nonspecific agents. Data presented in Table 5 suggest some small, statistically insignificant, differences in the releasing capacity of PHA, PMA, and fMLP. PHA and PMA presented similar stimulating capacity, whereas the cytokine amounts released by fMLP were slightly lower. In a pilot study, data of which are not shown, we have compared the stimulating capacity of these agents in various concentrations. The concentrations used in the present study were most effective in the cytokine release from the isolated blood cells, whereas the higher concentrations were not significantly more effective. 

Another important factor influencing the conclusions drawn from various studies is the time schedule of the material collection. The data presented in Table 6 show clearly that the significant changes in the concentrations of particular cytokines, both in plasma and in supernatants, appear 24–56 hours after the allergen challenge, and not before or shortly after the challenge. This fact would stress the importance of repeated recording of the investigated parameters, in this case cytokine concentrations, related to a well-defined and controlled event, such as bronchial allergen challenge. This is the only method to follow the dynamic aspects of the certain parameter changes. The single cytokine determination, related to a certain time interval only, seems to us to be of a limited value and can be accepted only for the screening purposes.

A complicating factor of the cytokine studies is a dearth of comprehensive data concerning the cytokine and chemokine profiles in peripheral blood, BAL fluid, or sputum of healthy subjects, which could be used as reference standards [13, 33, 47, 48]. The prechallenge serum as well as supernatant concentrations of cytokines recorded in DYAR patients did not differ significantly from healthy control subjects, whereas they differed from the values measured in patients developing IAR, LAR, or DLAR (Tables 4 and 5). The cytokine profiles recorded during the DYAR [45] differed also from those measured by other investigators in patients developing IAR, LAR, or DLAR [15, 16, 18, 25]. These facts would suggest involvement of different immunologic mechanisms in the DYAR and those underlying the other asthmatic response types.

The results of this study are consistent with the results of our previous studies [40, 41, 44, 45], especially with significant changes in the Th_1_/Th_2_ ratio in peripheral blood in favour of Th_1 _cells, increased intracellular concentrations of IFN-*γ* and IL-2, but not those of IL-4 or IL-5, increased blood leukocyte, neutrophil, and monocyte, but not eosinophil, counts, increased plasma concentrations of LTB_4_ and MPO, and serum concentration changes of various soluble adhesion molecules during the DYAR. 

These results would suggest involvement of the cell-mediated hypersensitivity upon participation of Th_1 _lymphocytes (IL-2, IFN-*γ*, GM-CSF, and TGF-*β*), neutrophils (IL-8, G-CSF, and TNF-*α*), monocytes (IL-18, TNF-*α*, and TGF-*β*) and probably also NK cells (IL-2, IFN-*γ*, IL-8, G-CSF, GM-CSF, and TGF-*β*) in the clinical DYAR. An additional role of macrophages (IL-8, IL-13, IL-18, TNF-*α*, GM-CSF, and G-CSF), epithelial and endothelial cells (IL-8, IL-18, and TNF-*α*) in the immunologic mechanisms leading to the development of the DYAR cannot be even excluded. However, since most of the cytokines are not exclusive products of only one cell type, but they can usually be produced by various cell types and lineages, the determination of the role of particular cell type by means of cytokine profiles becomes to be relatively complicated issue. Moreover, the number of cytokines investigated was relatively limited and some other, even important, cytokines, such as IL-17, IL-10 superfamily, and TNF superfamily members have not been included in the current study, either for technical reasons or with respect to the lack of the suitable commercial ELISA kits for these cytokines at the time of this study. This aspect can be considered as a certain deficit of the current study. Nevertheless, more concurrent investigations, including more cytokines, have to be performed to clarify the DYAR and the underlying immunologic mechanisms. 

## Figures and Tables

**Table 1:** Characteristics of the patients and control subjects.

	Patients DYAR *n* = 24	Control subjects			
		Patients with asthma			Healthy subjects
		IAR	LAR	DLAR	*n* = 26
		*n* = 21	*n* = 17	*n* = 18	
Age (years)	31 ± 4	30 ± 5	33 ± 3	28 ± 6	30 ± 6
Gender (M/F)	11/13	9/12	8/9	7/11	14/12
Disease history (years)	4.3 ± 2.1	4.5 ± 2.3	5.5 ± 1.4	6.3 ± 1.0	0
Asthmatic attacks per month	3 ± 1	3 ± 1	4 ± 1	4 ± 2	0
FEV_1_ (% predicted)	94.7 ± 7.8	95.5 ± 6.3	92.8 ± 5.4	96.1± 5.7	100.7 ± 3.6
FVC (% predicted)	97.5 ± 5.8	98.6 ± 3.7	100.3 ± 4.9	101.1 ± 6.0	103.9 ± 7.0
Blood leukocyte count (×10^9^/L)°	10.5 ± 0.6^+^	7.1 ± 0.6	8.0 ± 0.9	8.3 ± 0.5	6.5 ± 0.4
Blood neutrophil count (×10^9^/L)°°	7.1 ± 2.5^+^	5.5 ± 2.0	5.7 ± 2.1	6.0 ± 0.5	5.4 ± 1.6
Blood eosinophil count (×10^6^/L)°°°	311 ± 30	527 ± 65*	513 ± 41*	538 ± 55*	235 ± 22
Bronchial histamine threshold^□^					
≤2.0 mg/mL	0	3	2	2	0
4.0 mg/mL	2	5	1	3	0
8.0 mg/mL	3	5	4	4	0
16.0 mg/mL	6	4	5	5	0
32.0 mg/mL	4	2	3	3	2
>32.0 mg/mL	9	2	2	1	24
BPT positive/total^□□^	1/1-2	1/2	1/2	1/3	0
Ratio Th_1_/Th_2_ (%) in blood^●^	8.9 ± 2.2^+^	6.2 ± 3.0	6.8 ± 2.7	6.5 ± 2.4	7.1 ± 2.1
IFN-*γ* (pg/mL)—PBMC^●^	364 ± 45^+^	192 ± 40	189 ± 61	181 ± 55	221 ± 43
IL-4 (pg/mL)—PBMC^●^	17.9 ± 5.3	24.3 ± 5.4^+^	21.8 ± 4.1	23.4 ± 3.6^+^	18.4 ± 4.5

DYAR: delayed asthmatic response; IAR: immediate asthmatic response; LAR: late asthmatic response; DLAR: dual late asthmatic response; asthma: bronchial asthma; values = mean ± SD; statistical significance as compared with healthy control subjects: *P < 0.05; ^+^ *P* ≤ 0.05 (borderline); °normal value = 4.0 − 10 × 10^9^/L; °°normal value = 2.0 − 7.2 × 10^9^/L; °°°normal value ≤300 × 10^6^/L; ^□^normal value < 32.0 mg/mL; ^□□^BPT: bronchial provocation tests with allergens (per patient = positive/total); PBMC: peripheral blood mononuclear cells; ^●^after stimulation with PMA (Phorbol 12-myristate 13-acetate).

**Table 2:** Survey of the single diagnostic parameters.

	Patients *n* = 24	Control subjects			
		Bronchial asthma patients			Healthy subjects
		IAR	LAR	DLAR	*n* = 26
		*n* = 21	*n* = 17	*n* = 18	
Positive skin response (i.c.)					
Immediate	10	19	5	9	0
Late	2	2	12	9	0
Delayed	12	0	0	0	0
Increased total IgE in serum*	0	9	4	8	0
Increased total IgG in serum***	0	0	10	7	0
Increased IgG sub-classes in serum^+^					
IgG_1_	0	0	2	1	0
IgG_2_	0	0	0	0	0
IgG_3_	0	0	4	2	1
IgG_4_	0	0	5	2	0
Increased total IgM in serum^++^	0	0	0	0	0
Increased total IgA in serum^+++^	0	0	0	0	0

(i.c.): intracutaneous tests; *total IgE in the serum (PRIST): normal value = <500 IU/mL;**positive specific IgE in the serum (RAST) = >0.70 U/mL (= more than class 1); ***total IgG in the serum (single radial immunodiffusion = Mancini technique and ELISA): normal value = <15.0 g/L; ^+^IgG_1_ < 5.0 g/L; IgG_2_ < 2.6 g/L; IgG_3_ < 0.4 g/L; IgG_4_ < 0.5 g/L; ^++^IgM = <3.8 g/L (<1.5); ^+++^IgA = <4.0 g/L (<3.2).

**Table 3:** Allergens caused particular types of asthmatic response.

Allergen	Concentration	DYAR	IAR	LAR	DLAR	Healthy subjects
	BU/mL	*n* = 24	*n* = 21	*n* = 17	*n* = 18	*n* = 26
*Dermatophagoides pteronyssinus *	1000	5	7	4	6	0
*Dermatophagoides farinae *	1000	1	0	2	1	0
Animal danders						
Dog	3000	2	3	2	1	0
Cat	1000	2	1	1	2	0
Horse	2000	0	0	1	0	0
Hamster	2000	0	1	0	0	0
*Aspergillus fumigatus *	1000	1	0	0	1	0
Pollen						
Grass mix I	1000	5	4	3	2	0
Grass mix II	1000	4	2	2	0	0
Tree mix	3000	1	1	0	1	0
Weed mix	1000	1	0	0	2	0
Birch	1000	1	1	1	1	0
Poplar	2000	1	0	0	1	0
Ragweed giant	1000	0	1	1	0	0

BU/mL: biologic units per 1 mL.

Grass pollen mix I: *Dactylis glomerata*, *Lolium perenne*, *Phleum pratensis*, and *Poa pratensis*.

Grass pollen mix II: *Festuca pratensis*, *Holcus lanatus*, *Agrostis alba*, and *Anthoxanthum odoratum*.

Tree pollen mix: *Betula pendula*, *Corylus avellana*, *Juniperus communis*, and *Salix alba*.

Weed pollen mix: *Artemisia vulgaris*, *Plantago lanceolata*, *Rumex acetosa*, and *Taraxacum officinale*.

**Table 4:** Single determination of cytokine concentrations in the (nonstimulated) serum (pg/mL).

	Patients DYAR *n* = 24	Control subjects			
		BA patients			Healthy subjects
		IAR	LAR	DLAR	*n* = 26
		*n* = 21	*n* = 17	*n* = 18	
IL-1*β*	1.4 ± 0.2	1.9 ± 0.7	1.8 ± 0.6	1.7 ± 0.4	1.8 ± 0.2
IL-2	4.8 ± 1.6^+^	<3.0	<3.0	3.0 ± 0.3	3.1 ± 0.3
IL-3	<31.2	<31.2	<31.2	<31.2	<31.2
IL-4	3.4 ± 0.9	5.9 ± 2.2^+^	4.6 ± 1.0	5.1 ± 0.4^ +^	3.5 ± 0.3
IL-5	3.9 ± 1.0	7.1 ± 1.2*	4.5 ± 0.6	4.9 ± 1.1	4.0 ± 0.7
IL-6	<4.0^+^	5.8 ± 1.1	5.3 ± 0.4	6.7 ± 1.5*	5.1 ± 0.4
IL-7	1.4 ± 0.3	1.9 ± 0.8	1.3 ± 0.5	1.7 ± 1.0	1.2 ± 0.3
IL-8	6.1 ± 1.2	6.0 ± 1.4	9.5 ± 2.1*	8.3 ± 1.5^+^	5.3 ± 0.6
IL-10	3.7 ± 0.3	4.0 ± 0.6	6.2 ± 0.5	7.1 ± 0.6^+^	4.1 ± 1.0
IL-12p40	7.6 ± 2.1	6.9 ± 1.8	8.1 ± 4.2	9.8 ± 2.1^+^	7.9 ± 1.4
IL-12p70	8.9 ± 1.1	9.6 ± 1.3	9.4 ± 0.5	9.3 ± 1.0	9.2 ± 2.0
IL-13	<3.0	4.7 ± 0.5^+^	3.3 ± 0.2	3.9 ± 0.4	<3.0
IL-18	10.5 ± 0.4	13.8 ± 0.9^+^	17.0 ± 2.3*	14.8 ± 1.5*	10.0 ± 2.1
IFN-*γ*	2.9 ± 0.3^+^	<1.0	<1.0	<1.0	<1.0
GM-CSF	<3.0^+^	5.1 ± 1.6	5.3 ± 0.8	4.6 ± 0.7	3.8 ± 0.5
G-CSF	<0.8	<0.8	<0.8	<0.8	<0.8
TNF-*α*	6.8 ± 0.5	7.2 ± 1.1	8.3 ± 0.7^+^	5.5 ± 1.0	6.4 ± 0.5
TGF-*β*	12.3 ± 4.0	12.9 ± 2.1	17.1 ± 5.0^+^	14.0 ± 2.3	12.5 ± 4.0

Values = mean ± SEM; Statistical significance as compared with healthy subject values: *P < 0.05; ^+^ *P* ≤ 0.05 (borderline).

DYAR: delayed asthmatic response; IAR: immediate asthmatic response; LAR: late asthmatic response; DLAR: dual late asthmatic response.

**Table 5:** Mean values of cytokine concentrations in the supernatants (pg/mL) released from peripheral blood cells stimulated “*in vitro*” with various nonspecific stimulating agents.

		Control subjects			
	DYAR, *n* = 24	Asthmatics			Healthy subjects
	PHA/PMA/fMLP	IAR, *n* = 21	LAR, *n* = 17	DLAR, *n* = 18	*n* = 26
		PHA/PMA/fMLP	PHA/PMA/fMLP	PHA/PMA/fMLP	PHA/PMA/fMLP
IL-1*β*	7.1/7.4/6.8	7.5/7.3/7.0	7.4/7.7/7.5	8.2/8.1/8.4	8.5/8.3/8.0
IL-2	4.2/3.9/4.0*	<3.0	<3.0/3.2/<3.0	<3.0	<3.0
IL-3	<31.2	<31.2	33.2/33.5/<31.2^+^	<31.2	<31.2
IL-4	19.8/21.2/20.0	33.8/35.9/34.7*	24.0/23.5/23.9	31.0/32.8/33.0*	21.6/22.9/21.8
IL-5	5.1/6.0/5.7	8.7/9.5/9.3*	9.4/10.6/9.8*	9.5/11.0/9.7*	4.9/5.0/4.3
IL-6	4.6/5.0/5.2	6.4/7.1/7.2*	4.7/5.2/5.0	7.3/7.2/8.0*	4.0/4.8/3.7
IL-7	3.3/4.2/4.1	8.9/10.0/9.4*	3.5/3.5/3.8	4.1/4.4/3.4	3.9/3.5/3.6
IL-8	9.6/10.3/9.0^+^	7.4/7.1/7.9	10.5/11.0/10.6*	9.5/12.1/11.3*	8.2/7.0/6.5
IL-10	8.1/9.0/8.5	12.6/12.2/12.7*	8.3/7.6/7.9	8.1/8.0/7.9	7.7/8.4/8.2
IL-12p40	9.8/10.5/10.5	13.9/14.4/14.2^+^	15.6/15.0/15.3*	14.2/16.7/16.5*	10.1/9.6/9.5
IL-12p70	13.0/14.9/13.2^+^	14.0/14.8/14.6^+^	10.3/9.9/10.1	12.0/12.6/11.7	11.5/11.9/11.8
IL-13	3.8/<3.0/<3.0	10.1/10.7/9.8*	9.9/10.3/10.0*	11.0/11.5/10.8*	3.4/4.0/<3.0
IL-18	19.6/20.0/20.0*	14.1/13.9/13.7	15.2/15.5/15.0	16.1/15.7/15.5	15.9/16.5/14.2
IFN-*γ*	373/395/350*	199/207/183	191/212/205	213/218/211	224/237/255
GM-CSF	10.4/10.5/9.6	12.7/14.0/13.9*	13.5/14.3/13.8*	15.4/15.0/14.6*	10.0/9.4/7.0
G-CSF	7.0/7.4/6.8*	3.9/4.5/4.5	3.7/3.5/3.1	4.0/4.4/3.6	4.1/3.5/3.2
TNF-*α*	11.7/12.9/11.3^+^	8.5/8.9/9.1	10.4/11.7/11.6^+^	7.8/7.5/7.6	9.0/8.8/7.9
TGF-*β*	16.8/17.5/17.4	17.5/17.1/18.0	20.4/22.6/21.9^+^	23.0/23.7/22.8*	18.0/18.2/16.7

DYAR: delayed asthmatic response; IAR: immediate asthmatic response; LAR: late asthmatic response; DLAR: dual late asthmatic response. Values = means ± SEM. Statistical significance as compared with healthy subject values: *P < 0.05; ^+^ *P* ≤ 0.05 (borderline). PHA: Phytohemagglutin in (10 *μ*g/mL); PMA: Phorbol 12-myristate 13-acetate (50 ng/mL); fMLP: N-formylmethionyl-leucyl-phenylalanine (1 *μ*mol/L).

**Table 6:** Nonstimulated plasma concentrations and concentrations of cytokines in supernatants released by cells stimulated “*in vitro*” with PMA (mean ± SEM), during DYARs and PBS control tests.

Patients, *n* = 24	Before the challenge	After the challenge (hrs)						
		1	12	24	36	48	56	72
IL-1*β* (pg/mL)								
DYAR								
p	1.6 ± 0.5	1.5 ± 0.4	2.0 ± 1.1	3.3 ± 1.0	5.9 ± 0.3^+^	6.0 ± 0.7^+^	3.1 ± 0.4	2.7 ± 0.6
c	7.3 ± 1.1	8.4 ± 0.5	9.6 ± 1.0	9.2 ± 0.7	8.8 ± 0.6	9.3 ± 0.2	9.0 ± 1.0	8.6 ± 0.7
PBS								
p	1.5 ± 0.2	2.0 ± 0.7	3.0 ± 0.5	2.4 ± 0.3	2.2 ± 0.6	2.4 ± 0.8	2.5 ± 0.7	1.9 ± 0.4
c	7.4 ± 0.8	7.1 ± 0.2	8.2 ± 0.8	8.9 ± 0.4	9.4 ± 1.1	9.0 ± 0.6	8.5 ± 0.2	8.3 ± 0.5

IL-2 (pg/mL)								
DYAR								
p	3.5 ± 0.5	3.5 ± 0.3	5.2 ± 0.7^+^	7.8 ± 0.6*	7.5 ± 1.1*	6.9 ± 0.4*	4.3 ± 0.5	3.6 ± 0.3
c	3.7 ± 0.4	5.2 ± 0.1	8.6 ± 1.0*	9.3 ± 0.1*	8.0 ± 0.7*	9.4 ± 0.6*	6.7 ± 0.2*	3.8 ± 0.5
PBS								
p	3.3 ± 0.2	3.6 ± 0.5	3.4 ± 0.3	<3.0	3.9 ± 0.5	4.0 ± 0.7	<3.0	<3.0
c	4.0 ± 0.8	5.0 ± 0.9	7.1 ± 0.4	6.0 ± 0.3	4.8 ± 0.6	5.3 ± 0.2	4.9 ± 0.4	4.5 ± 0.4

IL-3 (pg/mL)								
DYAR								
p	<31.2	<31.2	<31.2	<31.2	32.2 ± 0.5	31.7 ± 0.6	32.0 ± 0.4	<31.2
c	34.2 ± 2.0	36.7 ± 2.4	38.0 ± 3.9	35.5 ± 1.2	37.6 ± 2.7	35.1 ± 1.5	36.9 ± 1.8	34.7 ± 1.0
PBS								
p	<31.2	31.2 ± 0.0	<31.2	31.5 ± 0.2	<31.2	<31.2	31.3 ± 0.1	<31.2
c	32.5 ± 1.8	35.3 ± 1.8	35.9 ± 1.2	37.4 ± 0.9	36.5 ± 1.4	33.7 ± 1.3	35.5 ± 2.0	35.2 ± 0.6

IL-4 (pg/mL)								
DYAR								
p	4.5 ± 0.8	4.0 ± 0.5	5.4 ± 0.6	5.0 ± 1.0	4.6 ± 0.4	5.5 ± 1.2	4.8 ± 0.5	4.2 ± 0.4
c	23.7 ± 1.6	25.0 ± 2.7	24.3 ± 1.9	22.0 ± 2.3	23.6 ± 0.5	22.9 ± 0.8	23.4 ± 1.1	23.1 ± 0.5
PBS								
p	3.9 ± 0.5	3.8 ± 0.6	4.2 ± 1.1	4.5 ± 0.6	5.4 ± 0.3	3.9 ± 0.6	4.0 ± 1.0	4.1 ± 0.5
c	22.9 ± 1.1	21.7 ± 1.0	22.6 ± 0.8	23.4 ± 1.5	22.7 ± 1.6	22.2 ± 1.0	21.6 ± 0.9	22.5 ± 1.1

IL-5 (pg/mL)								
DYAR								
p	4.1 ± 0.8	4.9 ± 0.9	4.5 ± 1.1	4.3 ± 0.6	4.5 ± 0.5	5.0 ± 1.0	4.4 ± 0.7	4.6 ± 0.4
c	6.9 ± 1.0	7.7 ± 0.8	7.5 ± 1.3	8.0 ± 0.4	7.1 ± 0.7	7.3 ± 2.1	7.6 ± 1.4	7.4 ± 0.9
PBS								
p	3.8 ± 0.5	3.7 ± 0.6	4.3 ± 0.5	4.1 ± 1.5	3.9 ± 0.4	4.8 ± 1.0	4.2 ± 0.9	4.5 ± 0.6
c	6.5 ± 0.3	7.5 ± 1.4	8.0 ± 0.7	7.9 ± 1.1	7.2 ± 0.6	7.2 ± 0.4	7.3 ± 0.5	7.6 ± 1.0

IL-6 (pg/mL)								
DYAR								
p	<4.0	<4.0	4.5 ± 04	<4.0	4.3 ± 0.2	<4.0	<4.0	<4.0
c	5.7 ± 1.1	7.8 ± 0.9	8.1 ± 1.3	8.6 ± 1.7	8.4 ± 0.6	7.9 ± 1.0	7.5 ± 0.4	7.0 ± 0.8
PBS								
p	<4.0	<4.0	<4.0	<4.0	4.7 ± 0.5	<4.0	4.2 ± 0.7	4.1 ± 0.3
c	5.9 ± 0.5	6.1 ± 1.3	7.2 ± 1.0	7.9 ± 2.3	8.0 ± 1.1	7.4 ± 0.3	7.7 ± 0.8	7.5 ± 0.5

IL-7 (pg/mL)								
DYAR								
p	1.7 ± 0.2	<0.1*	0.7 ± 0.2	0.3 ± 0.1^+^	<0.1*	<0.1*	<0.1*	0.2 ± 0.1^+^
c	4.3 ± 0.5	5.7 ± 1.0	6.2 ± 0.8	6.5 ± 0.4	5.9 ± 1.0	6.6 ± 0.7	6.3 ± 0.4	5.8 ± 0.3
PBS								
p	2.0 ± 0.1	2.1 ± 0.8	1.7 ± 0.4	1.3 ± 0.5	2.5 ± 0.7	2.1 ± 0.4	1.7 ± 0.9	2.2 ± 1.1
c	4.6 ± 0.3	4.9 ± 0.5	5.5 ± 1.0	5.8 ± 0.9	6.2 ± 1.3	6.3 ± 1.1	5.6 ± 0.7	5.3 ± 0.6

IL-8 (pg/mL)								
DYAR								
p	6.8 ± 1.0	7.0 ± 1.1	7.2 ± 0.5	6.7 ± 0.8	6.5 ± 0.4	11.5 ± 0.9*	11.8 ± 1.0*	7.0 ± 0.5
c	12.2 ± 2.1	12.6 ± 1.8	13.5 ± 2.0	16.9 ± 2.6*	17.6 ± 1.7*	16.8 ± 2.0*	12.9 ± 1.8	12.8 ± 1.4
PBS								
p	7.2 ± 1.8	7.5 ± 2.0	6.8 ± 1.2	7.3 ± 0.5	7.1 ± 0.6	6.9 ± 0.8	7.3 ± 0.5	7.0 ± 0.7
c	11.4 ± 0.6	12.5 ± 1.0	12.9 ± 1.5	13.6 ± 1.8	14.0 ± 1.9	13.5 ± 1.1	13.5 ± 2.1	13.2 ± 1.0

IL-10 (pg/mL)								
DYAR								
p	3.5 ± 0.4	3.6 ± 0.4	3.8 ± 1.0	5.5 ± 0.4^+^	4.1 ± 1.0	3.4 ± 0.7	3.8 ± 0.9	4.1 ± 0.6
c	10.9 ± 2.2	10.3 ± 0.6	11.5 ± 0.8	11.2 ± 0.3	12.4 ± 1.3	11.7 ± 0.5	11.0 ± 1.0	11.3 ± 0.4
PBS								
p	<3.0	<3.0	<3.0	<3.0	<3.0	<3.0	3.2 ± 0.1	<3.0
c	9.5 ± 0.6	10.0 ± 0.3	9.8 ± 0.6	10.9 ± 1.1	11.2 ± 0.7	10.8 ± 0.4	9.9 ± 0.3	10.2 ± 0.5

IL-12p40 (pg/mL)								
DYAR								
p	8.0 ± 2.0	9.0 ± 3.2	8.1 ± 1.1	8.8 ± 1.9	8.2 ± 0.6	7.8 ± 0.7	8.5 ± 0.4	8.1 ± 1.0
c	10.7 ± 0.6	11.8 ± 1.3	12.5 ± 2.2	11.6 ± 0.8	10.5 ± 1.0	11.4 ± 0.9	11.0 ± 1.1	11.3 ± 0.6
PBS								
p	7.7 ± 1.3	7.9 ± 0.4	7.6 ± 0.5	7.9 ± 1.3	7.5 ± 1.0	8.0 ± 0.6	7.4 ± 0.9	7.9 ± 0.8
c	11.0 ± 1.4	12.1 ± 2.5	12.8 ± 2.0	11.9 ± 0.7	12.3 ± 2.5	11.6 ± 0.4	11.9 ± 1.8	11.7 ± 1.2

IL-12p70 (pg/mL)								
DYAR								
p	9.0 ± 1.9	10.3 ± 2.0	11.5 ± 2.1	19.3 ± 3.2*	17.9 ± 3.0*	16.8 ± 1.3*	9.9 ± 1.0	10.2 ± 0.5
c	15.5 ± 2.8	15.4 ± 1.9	18.2 ± 3.0	24.5 ± 2.4*	25.8 ± 1.6*	27.9 ± 3.5*	27.2 ± 2.8*	16.9 ± 1.4
PBS								
p	10.4 ± 2.1	10.1 ± 1.0	10.9 ± 1.2	10.0 ± 1.3	9.8 ± 0.8	9.9 ± 1.0	10.1 ± 0.7	9.6 ± 1.2
c	16.0 ± 3.3	16.2 ± 0.8	16.9 ± 1.3	18.0 ± 0.5	17.7 ± 1.4	18.1 ± 0.6	18.5 ± 1.1	17.7 ± 1.5

IL-13 (pg/mL)								
DYAR								
p	<3.0	<3.0	4.1 ± 0.5	<3.0	3.7 ± 0.4	6.9 ± 0.7*	8.2 ± 0.9*	7.3 ± 1.0*
c	6.6 ± 0.3	7.1 ± 1.0	8.4 ± 0.7^+^	8.1 ± 0.9	9.8 ± 1.1*	10.2 ± 1.3*	8.5 ± 1.1^+^	6.9 ± 0.5
PBS								
p	<3.0	<3.0	<3.0	<3.0	<3.0	<3.0	3.1 ± 0.1	<3.0
c	7.0 ± 0.8	7.5 ± 1.2	7.1 ± 0.9	8.3 ± 1.5	8.6 ± 2.0	8.1 ± 1.4	7.9 ± 0.5	7.6 ± 0.8

IL-18 (pg/mL)								
DYAR								
p	11.0 ± 1.7	11.4 ± 2.3	12.3 ± 2.0	11.5 ± 1.0	13.9 ± 0.5^+^	14.6 ± 1.0*	14.8 ± 0.5*	10.5 ± 0.8
c	20.1 ± 1.3	19.6 ± 1.0	23.4 ± 0.8	26.7 ± 1.5^+^	27.5 ± 1.2*	28.0 ± 2.6*	30.1 ± 4.0*	26.3 ± 2.7
PBS								
p	11.3 ± 2.9	12.0 ± 1.5	11.1 ± 0.9	12.0 ± 0.2	11.0 ± 0.6	10.8 ± 1.0	10.4 ± 0.7	11.2 ± 0.4
c	19.7 ± 0.8	20.4 ± 2.0	22.7 ± 2.5	21.9 ± 1.4	22.6 ± 0.8	22.8 ± 0.7	21.8 ± 1.4	20.9 ± 1.6

IFN-*γ* (pg/mL)								
DYAR								
p	2.4 ± 0.3	2.7 ± 0.7	4.2 ± 0.6	14.1 ± 2.0*	17.9 ± 2.1*	15.7 ± 1.8*	8.0 ± 1.0*	2.9 ± 0.5
c	361 ± 108	383 ± 115	405 ± 111	475 ± 131*	489 ± 125*	503 ± 89*	466 ± 103*	401 ± 114
PBS								
p	2.1 ± 0.2	2.9 ± 0.3	2.3 ± 1.1	2.4 ± 0.3	2.2 ± 0.9	2.1 ± 0.4	2.3 ± 0.2	2.5 ± 0.6
c	367 ± 91	354 ± 68	288 ± 94	339 ± 102	321 ± 113	344 ± 116	352 ± 97	370 ± 105

GM-CSF (pg/mL)								
DYAR								
p	<3.0	3.5 ± 0.3	4.2 ± 0.6^+^	5.1 ± 1.3*	4.9 ± 0.7^+^	3.1 ± 0.1	3.0 ± 0.0	<3.0
c	10.2 ± 0.4	11.8 ± 0.9	13.5 ± 1.7	12.8 ± 1.0	13.1 ± 2.2	12.5 ± 0.8	11.4 ± 1.0	11.2 ± 0.5
PBS								
p	<3.0	<3.0	<3.0	<3.0	3.0 ± 0.0	<3.0	<3.0	<3.0
c	10.7 ± 0.6	10.4 ± 0.5	12.3 ± 1.0	11.9 ± 1.2	11.0 ± 1.4	11.3 ± 0.6	10.8 ± 1.2	11.5 ± 1.0

G-CSF (pg/mL)								
DYAR								
p	<0.8	1.7 ± 0.4	6.5 ± 1.8*	6.9 ± 0.7*	5.4 ± 0.9*	1.5 ± 0.4	<0.8	<0.8
c	7.1 ± 0.6	8.4 ± 1.0	8.9 ± 0.7^+^	9.5 ± 2.2^+^	9.0 ± 1.6^+^	8.6 ± 0.7	7.5 ± 1.2	7.8 ± 1.1
PBS								
p	<0.8	<0.8	<0.8	1.0 ± 0.1	0.9 ± 0.1	<0.8	<0.8	<0.8
c	7.5 ± 0.4	7.2 ± 0.5	7.6 ± 0.9	8.7 ± 1.3	8.2 ± 0.5	8.0 ± 1.3	7.9 ± 1.0	7.4 ± 0.6

TNF-*α* (pg/mL)								
DYAR								
p	7.1 ± 2.0	8.0 ± 1.7	7.4 ± 0.8	8.2± 1.2	14.5 ± 2.0*	12.4 ± 1.6*	13.0 ± 0.5*	8.0 ± 0.7
c	12.9 ± 1.3	13.4 ± 0.8	16.6 ± 1.1^+^	17.3 ± 0.5*	17.9 ± 0.7*	18.0 ± 2.2*	17.5 ± 1.0*	15.4 ± 1.1
PBS								
p	6.7 ± 1.1	7.0 ± 0.5	6.9 ± 0.8	6.5 ± 0.3	7.4 ± 1.4	7.0 ± 1.0	7.2 ± 0.8	6.5 ± 0.3
c	13.2 ± 0.8	13.6 ± 1.0	14.1 ± 2.0	14.7 ± 1.8	15.0 ± 1.6	14.9 ± 2.1	15.1 ± 1.3	14.6 ± 0.5

TGF-*β* (pg/mL)								
DYAR								
p	11.0 ± 2.3	12.5 ± 2.3	24.5 ± 3.7*	21.1 ± 2.8*	19.0 ± 2.5*	18.4 ± 1.0*	11.3 ± 0.5	10.8 ± 0.3
c	17.2 ± 1.7	21.4 ± 3.1	29.5 ± 2.2*	34.3 ± 2.0*	30.1 ± 1.9*	24.8 ± 2.5^+^	18.7 ± 1.5	18.2 ± 0.9
PBS								
p	10.1 ± 1.2	10.5 ± 0.8	11.3 ± 0.2	10.8 ± 1.0	10.4 ± 0.7	11.0 ± 0.5	10.6 ± 0.7	11.5 ± 0.4
c	18.0 ± 1.1	18.8 ± 1.0	20.9 ± 0.8	19.4 ± 1.3	21.2 ± 1.0	20.5 ± 0.9	19.0 ± 1.0	19.3 ± 0.6

DYAR: delayed asthmatic response; PBS: phosphate buffered saline (control); PMA: phorbol 12-myristate 12-acetate.

Values of cytokines = means ± SEM; statistical significance of the cytokine concentrations as compared with their prechallenge (baseline) values: *P < 0.05, ^+^ *P* ≤ 0.05 (borderline); p: plasma; c: supernatants of cells stimulated with PMA.
